# Cardiovascular Outcomes in Children with Multisystem Inflammatory Syndrome Treated with Therapeutic Plasma Exchange

**DOI:** 10.3390/children9111640

**Published:** 2022-10-27

**Authors:** Tunç Tunçer, Fatih Varol

**Affiliations:** 1 Department of Pediatrics, Division of Pediatric Cardiology, Saglik Bilimleri University Sancaktepe Sehit Prof. Dr. Ilhan Varank Training and Research Hospital, 34785 Istanbul, Turkey; 2 Department of Pediatrics, Division of Pediatric Intensive Care Unit, Saglik Bilimleri University Sancaktepe Sehit Prof. Dr. Ilhan Varank Training and Research Hospital, 34785 Istanbul, Turkey

**Keywords:** multisystem inflammatory syndrome, therapeutic plasma exchange, cardiovascular outcomes

## Abstract

Background: Multisystem inflammatory syndrome in children is a new, rare, post-infectious complication of SARS-CoV-2 infection in children. The aim of this study was to display the role of therapeutic plasma exchange on cardiovascular outcomes in children with multisystem inflammatory syndrome. Methods: This study included children who met the diagnostic criteria for multisystem inflammatory syndrome and who were admitted to the intensive care unit. This is a prospective single-center study conducted between August 2020 and September 2021. Subjects had cardiac involvement which was defined as elevated troponin I, abnormal electrocardiographic and echocardiographic findings. Patients were followed by a pediatric cardiologist throughout the intensive care unit stay and after discharge for 12 months. Patients were divided into two groups which received therapeutic plasma exchange and which did not. Results: 38 children were included in this study. There were 16 patients in the no plasma exchange group and 22 patients in the plasma exchange group. The two groups were similar in age, sex, leucocyte, thrombocyte count, neutrophil percentage, hemoglobin, C-reactive protein, erythrocyte sedimentation rate, alanine aminotransferase, albumin, ferritin, fibrinogen, D-dimer, IL-6, troponin I, number of electrocardiographic abnormalities and patients with mitral valve regurgitation detected at admission (*p* > 0.05). There was no significant difference between the two groups in terms of duration of normalization of electrocardiographic abnormalities and disappearance of mitral valve regurgitation (*p* > 0.05). Duration of normalization of troponin I (2, IQR 1–4, versus 5, IQR 3–9 days; *p* = 0.044) and length of hospital stay (7, IQR 6–10, versus 13, 8–20 days; *p* = 0.001) was longer in the plasma exchange group. Conclusions: We did not observe any significant improvement in children having undergone plasma exchange as compared to children who did not. On the opposite, their length of hospital stay and time to troponin I normalisation were even longer. Some baseline differences in cardiac attainment severity may partly explain this finding.

## 1. Introduction

In children exposed to the severe acute respiratory syndrome coronavirus 2 (SARS-CoV-2), a new disease with a severe inflammatory response has been identified on 26 April 2020. The new illness is known by two synonymous names: paediatric inflammatory multi-system syndrome associated with COVID-19 (PIMS-ts) and multisystem inflammatory syndrome in children (MIS-C) [1,2]. Although preliminary data revealed that previously healthy children were spared from severe symptoms, over time reports of mortalities emerged in children with multisystem inflammatory syndrome in children [3,4,5,6,7]. 

Multisystem inflammatory syndrome in children emerges 2–6 weeks after mostly asymptomatic or slightly symptomatic SARS-CoV-2 infection, indicating a delayed post-infectious response [8,9]. Cardiac abnormalities including abnormal cardiac enzymes, abnormal electrocardiograms, decreased systolic function, coronary artery abnormalities ranging from coronary dilation to giant aneurysms, mitral valve regurgitation, tricuspid valve regurgitation, aortic valve insufficiency, pericardial effusion, diastolic dysfunction, abnormal cardiac strain, and abnormal cardiac MRI have been detected in children with MIS-C [10,11,12]. Even though the majority of these anomalies resolved after short-term follow-up, more research is needed to determine the long-term negative cardiac outcomes [13]. The cornerstone of treatment for individuals with multisystem inflammatory syndrome in children includes intravenous immunoglobulins, glucocorticoids, anakinra, anticoagulants, and/or antiaggregant medications [14]. Information on therapeutic plasma exchange and plasmapheresis and its effect on cardiovascular outcomes are pretty scanty. Therefore, in this study, we aimed to investigate the role of therapeutic plasma exchange treatment in short and mid-term cardio-vascular outcomes of multisystem inflammatory syndrome in children.

## 2. Materials and Methods

### 2.1. Study Population and Protocol

The study has been approved by the institutional research ethics committee of Saglik Bilimleri University Prof. Dr. IlhanVarank Training and Research Hospital on 17 July 2020 (document number of 2020-233) before the study was started and has been conducted in accordance with the principles set forth in the Declaration of Helsinki. Informed consent for participation in the research study was obtained from the patients or patient’s parents before enrollment.

This is a prospective single-center study of 38 children treated for multisystem inflammatory syndrome in children at Sancaktepe Training and Research Hospital intensive care Unit in Istanbul between August 2020 and September 2021. The World Health Organization (WHO) and the Centers for Disease Control and Prevention (CDC) criteria were used to make the clinical diagnosis of multisystem inflammatory syndrome in children [2,15]. COVID-19 PCR and COVID-19 IgM and IgG results were obtained despite the fact that proof of SARS-CoV-2 infection was not required for inclusion. On intensive care unit admission age, sex, comorbidities, symptoms, clinical signs, leucocyte and thrombocyte counts, neutrophil percentage, hemoglobin, C-reactive protein (CRP), sedimentation rate, alanine aminotransferase, albumin, ferritin, fibrinogen, D-dimer, IL-6, NT-proBNP, procalcitonin, troponin I were recorded. Mode of mechanical ventilation, immunosuppressive treatment, systemic steroids, subcutaneous enoxaparin treatments, extracorporeal treatment and therapeutic plasma exchange treatments were recorded throughout the patients’ intensive care unit stay. A pediatric radiologist evaluated and documented the imaging results from the chest computed tomography and X-ray. Selected subjects had cardiac involvement which was defined as positive laboratory findings (elevated high-sensitivity troponin I), abnormal electrocardiographic and echocardiographic findings. Some patients had only elevated troponin levels and others had only abnormal electrocardiographic findings, while there were also patients who presented elevated troponin levels as well as abnormal electrocardiographic and echocardiography findings. Patients were monitored by a pediatric cardiologist throughout their intensive care unit stay. They were also followed at 4 weeks and/or at 3, 6, 9 and 12 months after admission. In order to assess the role of therapeutic plasma exchange, patients were divided into two groups; the plasma exchange (the PE group) received therapeutic plasma exchange while the no plasma exchange (the WPE group) group did not. 

Initial clinical evaluation included physical examination, vital signs, laboratory data, electrocardiogram, chest X-ray, computed tomography of the chest (if needed) and echocardiographic examination. At each follow-up visit, electrocardiogram and echocardiographic examination were performed. The patients’ routine data including demographics (age and sex), disease outcomes (length of stay), treatment (respiratory support and immunomodulatory agents), and laboratory data were obtained from the medical records.

### 2.2. Electrocardiographic Assessment

Patients were constantly monitored using bedside 3-lead electrocardiography device with 12-lead electrocardiography recorded every 24 h and additionally as needed during their intensive care unit stay. 12-lead electrocardiography was performed at all their follow-up visits. Low voltage on the electrocardiography was determined as QRS complexes of <0.5 mV in all limb leads and 1.0 mV in all precordial leads. ST-T changes were defined as J point elevation by >0.25 mV in boys or >0.15 mV in girls in leads V2–3 and >0.1 mV in all other leads; ST depression was determined as horizontal or downsloping ST segment ≥1 mV. 

### 2.3. Echocardiographic Assessment

Transthoracic echocardiography was performed using a Philips Affiniti 50 c echocardiography system (Philips Healthcare, Andover, MA, USA). Philips S4-2 Cardiac Sector Probe and Philips S8-3 Cardiac Sector Probe were used. All subjects were evaluated by the same pediatric cardiologist. M-mode, 2D, color, pulsed and continuous-wave Doppler, and tissue Doppler were employed as standard echo modes. The procedure was performed as needed during the patients intensive care unit stay as well as at all their follow-up visits. Absolute dimensions (millimeters), systolic function, and z-scores of the proximal coronary arteries were measured, including the left main coronary artery, left anterior descending coronary artery, left circumflex coronary artery, and right coronary artery. Reduced left ventricle systolic function was defined as fractional shortening < 28%. Coronary aneurysms were defined using z-scores per American Heart Association Kawasaki guidelines (small aneurysm z-scores ≥ 2.5 to <5.0, medium aneurysm z-score ≥ 5 to <10, and absolute dimension < 8 mm, and large aneurysm z-score ≥ 10, or absolute dimension ≥ 8 mm) [16].

### 2.4. Therapeutic Plasma Exchange Procedure

In our study therapeutic plasma exchange was used in cases of multi-organ failure caused by a cytokine storm associated with COVID-19, as well as unresponsiveness to other immunosuppressive treatments such as intravenous immunoglobulins and/ or steroids and liver dysfunction. Plasma exchange was performed by membrane separation. Prismaflex^®^ (Baxter International Inc., Deerfield, IL, USA) therapeutic plasma exchange 1000 and therapeutic plasma exchange 2000 sets were used. The amount of plasma was calculated as “estimated plasma volume (L) = 0.07 × weight (kg) × (1 − hematocrit)”. The replacement fluid was fresh frozen plasma. Saline 0.9% was used to prime the therapeutic plasma exchange circuit. Heparin (10–20 U/kg) was given every hour. Throughout this infusion, activated clotting time (ACT) was monitored at intervals of 170–220 s. During the therapeutic plasma exchange procedure, blood flow was adjusted to 2–6 mL/min/kg based on the patient’s weight. Vital signs were meticulously monitored and control blood samples were taken immediately before and after therapeutic plasma exchange.

### 2.5. Statistics

JASP (Jeffreys’s Amazing Statistics Program, version 0.14.1.0, Department of Psychological Methods, University of Amsterdam, The Netherlands) was used for statistical analysis. Normality was evaluated with Shapiro–Wilk tests and histograms. The data were expressed as mean, standard deviation, median, interquartile range, minimum, maximum, frequency and percentage. The comparison of the categorical variables was done with the Yate’s chi-squared test or Fisher’s exact test. Normally distributed continuous variables were compared with the Student’s *t*-test. A Mann–Whitney U test was used for continuous variables that did not have a normal distribution. Statistically significance was considered as a *p*-value of <0.05. This being an observational trial with opportunistic recruitment, a sample size calculation was not performed. However, a post-hoc power calculation, setting an alfa level of 0.05, was performed.

## 3. Results 

Thirty-eight children were included in this study. Ten of the 16 patients (62.5%) in the WPE group and 13 of the 22 patients (59%) in the PE group were male. The two groups were similar in age and sex (*p* > 0.05). Symptoms and signs of the WPE group at admission were fever (*n*: 14, 87.5%), abdominal pain (*n*: 7, 43.7%), vomiting (*n*: 7, 43.7%), diarrhea (*n*: 6, 37.5%), conjunctivitis (*n*: 5, 41.6%), lymphadenopathy (*n*: 4, 25%), red cracked lips (*n*: 2, 12.5%), hypotension (*n*: 2, 12.5%), headache (*n*: 1, 6.2%), cough (*n*: 1, 6.2%), tachypnea (*n*: 1, 6.2%), oliguria (*n*: 1, 6.2%), rash (*n*: 1, 6.2%), chest pain (*n*: 1, 6.2%), nausea (*n*: 1, 6.2%) and symptoms and signs of the PE group were fever (*n*: 21, 95.4%), abdominal pain (*n*: 16, 72.7%), vomiting (*n*: 16, 72.7%), diarrhea (*n*: 14, 63.6%), hypotension (*n*: 8, 36.3%), conjunctivitis (*n*: 6, 27.2%), lymphadenopathy (*n*: 6, 27.2%), cough (*n*: 3, 13.6%), shock (*n*: 3, 13.6%), red cracked lips (*n*: 1, 4.5%, chest pain (*n*: 1, 4.5%), nausea (*n*: 1, 4.5%) (Supplementary Table S1). There were no statistically significant differences in the presentation symptoms and signs in the two groups. Three patients had pre-existing comorbidities in the WPE group (epilepsy, chronic renal insufficiency, acute lymphoblastic leukemia) and two patients in the PE group (inflammatory bowel disease and hypertrophic cardiomyopathy). The respiratory system was involved in 13 (81.2%) of the patients in the WPE group and 19 (86.3%) of the patients in the PE group, diagnoses being supported by chest X-rays and chest computerized tomography findings consistent with COVID-19. Three system involvements were detected in two patients (12.5%) in the WPE group and in four patients (18.1%) in the PE group. Two patients (12.5%) in the WPE group and 9 patients (40.9%) in the PE group were admitted with hypotension, two of whom in PE were also in shock. Five patients (31%) were admitted with Kawasaki-like symptoms in the WPE group and six patients (27%) in the PE group (Supplementary Table S1). There were no significant differences between the two groups in terms of respiratory system involvement, admission with hypotension and Kawasaki-like symptoms (*p* > 0.05). Two patients (5.2%) died in the intensive care unit, one from each group and they are shown in Supplementary Table S1. The one in the WPE group, aged 10 years was being treated for acute lymphoblastic leukemia when diagnosed with multisystem inflammatory syndrome in children. Coronary artery dilation and left ventricular systolic dysfunction were detected in this patient. The other patient who died in intensive care unit after 17 days and who was in the PE group had also left ventricular systolic dysfunction at admission (Supplementary Table S3).

The PE group patients had significantly higher levels of NT-proBNP (*p* = 0.01) and procalcitonin (*p* = 0.035) at admission. However, there was no significant difference between the two groups at admission in terms of leucocyte, neutrophil, thrombocyte count, hemoglobin, C-reactive protein (CRP), sedimentation rate, alanine aminotransferase (ALT), albumin, ferritin, fibrinogen, D-dimer, IL-6 and troponin I (*p* > 0.05). A comparison of the demographics, clinical characteristics and laboratory values of the patients are summarized in Table 1 and Supplementary Tables S1 and S2. 

One patient (6.2%) in the WPE group had only electrocardiographic change in terms of cardiac involvement which was low voltage detected on the electrocardiography. Electrocardiographic changes were detected in only half of the patients in the WPE group and in the PE group which consisted of low voltage (18.7%), QT segment prolongation of median 458 milliseconds (12.5%) and T wave changes (6.2%) in the WPE group and low voltage (18.1%), QT segment prolongation of median 462 milliseconds (22.7%), first degree block (4.5%), ST segment depression/elevation (4.5%), left ventricular hypertrophy (4.5%) in the PE group. QT segment prolongation was not associated with any electrolyte disturbances like hypokalemia, hypocalcemia or hypomagnesemia in neither group. QTc values of patients with QT segment prolongation are shown in Table 2 and Supplementary Table S3. Electrocardiographic abnormalities of both groups returned to normal during the intensive care period and there was no significant difference between the two groups (*p* > 0.05) in terms of duration of normalization (Table 2).

Three patients (18.7%) in the WPE group and four patients (18.1%) in the PE group had only isolated elevated troponin in terms of cardiac involvement. Duration of normalization of troponin I in PE was significantly longer than the WPE group (Table 2).

Mild mitral valve regurgitation was detected in half of the patients in the WPE group and in 59% of the patients in the PE group at admission. There was no significant difference between the two groups in terms of the number of mild mitral valve regurgitation detected at admission and duration of disappearance (Table 2). Moderate mitral valve regurgitation detected in two patients in the WPE group and in the PE group changed to mild mitral valve regurgitation after 6 and 8 days respectively however it never disappeared during the entire follow-up. Three patients in the PE group had mild aortic regurgitation, two of whom had also mitral valve regurgitation and reduced left ventricular systolic function, which disappeared during their intensive care stay. It should be stressed that mild, central aortic regurgitation and mild mitral valve regurgitation are normal by definition, which do not need treatment and follow-up (Supplementary Table S3).

Reduced left ventricular systolic function (fractional shortening < 28%) was detected in 25% of the patients in the WPE group and 27% of the patients in the PE group at admission (Table 2). There was no significant difference between the two groups in terms of the number of reduced left ventricular systolic function detected at admission (Table 2). Two patients in the WPE group had coronary artery dilatations. One of them died due to complications of acute lymphoblastic leukemia. The other patients’ coronary artery dilatation of left main and left anterior descending artery resolved in six days without aneurysm formation. Two patients in the PE group had increased perivascular echogenicity of the left main coronary artery which resolved in five days, one patient in the PE group had coronary artery dilatation of right coronary artery which resolved in six days without aneurysm formation (Supplementary Table S3). The results of the post-hoc power calculation, setting an alfa level at 0.05, are as follows: for the (1) length of hospital stay 98%, (2) time to normalization of troponin 64%, (3) normalization of ECG abnormalities 63%, while the remaining comparisons had powers < 40%.

There was no significant difference (*p* > 0.05) between the two groups in terms of intravenous immunoglobulins, systemic steroids, subcutaneous enoxaparin treatments, invasive mechanical ventilatory support and follow-up time (Supplementary Table S4). However there was a significant difference between the two groups in terms of the days of duration of stay in pediatric intensive unit, with the PE group being longer than the WPE group (Table 2) (Supplementary Table S4).

Three patients (13.6%) in the PE group were placed on extracorporeal membrane oxygenation. There was no significant difference (*p* > 0.05) between the two groups in terms of patients placed on extracorporeal membrane oxygenation. They all had reduced left ventricular systolic function. Left ventricular systolic function of the two patients improved and they were successfully weaned; however, one of them was unfortunately successively diagnosed with brain death and died during the intensive care unit period (Supplementary Table S4). 

Three patients in the PE group (13.6%) received tocilizumab. All of these patients had reduced left ventricular systolic function at admission and two of them were also given the interleukin-1 receptor antagonist anakinra. Subject number seven in the PE group who received anakinra and tocilizumab died in the intensive care unit (Supplementary Table S1). Left ventricular systolic function of the other two patients recovered. One patient in the PE group took only anakinra without tocilizumab (Supplementary Table S4). 

In therapeutic plasma exchange, patients had 1 or 1.5 times their total plasma volume administered using fresh frozen plasma. Patients had a minimum of *n* = 2 and a maximum of *n* = 9 sessions, with a mean of 5.3 (median 5) sessions of therapeutic plasma exchange performed. Patients did not experience any side effects during or after the procedure.

## 4. Discussion

Plasma exchange is an extracorporeal blood purification technology that separates and removes pathological plasma from the patient’s blood while infusing a specific amount of solution or normal human plasma to eliminate pathogenic substances and reduce pathological damage [17]. Therapeutic plasma exchange may be able to stabilize critically ill or fast deteriorating patients and lower mortality by eliminating inflammatory cytokines, stabilizing the endothelium membrane, and resetting the hypercoagulable condition. C-reactive protein, interleukin-6, lactate dehydrogenase, and D-dimer levels are lower after therapeutic plasma exchange in critically ill adults with COVID-19. Oxygenation index improved, extubation rate increased, and all-cause mortality decreased [18,19,20]. Detailed reports of therapeutic plasma exchange in pediatric COVID-19 patients with multisystem inflammatory syndrome are scarce and the studies on the effects of therapeutic plasma exchange treatment on cardiovascular outcomes in children with multisystem inflammatory syndrome are even more limited.

Electrocardiographic changes were detected in only half of the patients in both groups in our study which is in line with the study of Atasayan et al. [21]. 24-h Holters were recorded in 61.2% of the patients, 50.8% of them displaying pathological findings.

Atay et al. investigated the role of therapeutic plasma exchange in multisystem inflammatory syndrome in 41 children [22]. They concluded that therapeutic plasma exchange may be effective in critically ill patients. In their review study consisting of adult patients, Krzych et al. evaluated the role of therapeutic plasma exchange treatment in severe COVID-19 cases [23]. They found that the procedure seemed to improve various secondary end-points such as PaO_2_/FiO_2_ ratio or biomarkers of inflammation; however, the effect of therapeutic plasma exchange on mortality remained unclear. This review also mentioned LDH, ferritin, IL-6, CRP, and D-dimers as biomarkers for predicting the severity of the disease. The median IL-6 values in our study were in line with those found in that systematic review. Although therapeutic plasma exchange is reserved for critically ill patients, the PE group in our study who needed therapeutic plasma exchange and extracorporeal membrane oxygenation were similar to the WPE group in terms of inflammatory markers and other laboratory values at baseline except for NT-proBNP and procalcitonin. However, it should be noted that some of them were unresponsive to intravenous immunoglobulins and steroid treatment which made them candidates for therapeutic plasma exchange. There was no significant difference between our two groups regarding reduced left ventricular systolic function and mild mitral valve regurgitation at admission. However, the PE group had significantly higher NT-proBNP, a marker known to correlate well with both clinical heart failure severity and prognosis, and procalcitonin, a marker of inflammation. The comparability of both groups is therefore intrinsically biased, and this (which might have disfavored the PE group) should be beard in mind while interpreting the results. It should be noted that severity score (SOFA & APACHE II) and cytokine levels (IL-6, C-reactive protein) can be used to discuss the indication for therapeutic plasma exchange therapy and to monitor response in COVID-19 patients [24]. Severity scores were not documented in our study for therapeutic plasma exchange treatment selection, one of the shortcomings of our study. Furthermore, regrettably, we only collected information on the number of patients with fractional shortening <28%, but no data on this continuous parameter of LV contractility was collected, hampering an appropriate comparison of this parameter with a test for continuous variables between the two groups. 

We detected significant difference in terms of procalcitonin and NT-proBNP at admission in our groups. It is evident that NT-proBNP is a useful tool in the diagnosis of heart failure, the assessment of clinical severity and follow-up of pediatric heart diseases [25]. Patients with high NT-proBNP values may be regarded as clinically more severe in terms of cardiac involvement. As stated above, the higher NT-proBNP in the PE group at baseline might theoretically have confounded or masked some potential beneficial effects of this therapy. Even though the perivascular (hyper) echogenicity in Kawasaki disease (which served as a basis to handle MIS-C patients, especially in its early “history” back in 2020) had been used in the past, it is not considered part of the definitions of coronary involvement any more in the most recent recommendations [16].

A further limitation is that severity scores were not documented in our study for therapeutic plasma exchange treatment selection. Besides, owing to the small number of patients, for some comparisons the power was very scarce, so that interpretability of results is limited. Furthermore, fractional shortening was used instead of ejection fraction, which is ideally estimated by the Simpson’s biplane method. Finally, the use of anakinra, tocilizumab and extracorporeal membrane oxygenation are potential confounding factors in our study.

All in all, although the small sample size of surviving subjects severely limits the interpretability of the results, in this study therapeutic plasma exchange did not seem to have considerable effects on cardiovascular outcomes. 

## 5. Conclusions

We did not detect differences in the time to LV systolic function normalization in patients treated with therapeutic plasma exchange. Furthermore and surprisingly, length of hospital stay and the duration of normalization of troponin I appeared to be even longer in the PE group. We concluded that therapeutic plasma exchange treatment does not provide a clinically relevant benefit. 

## 6. Limitations

Severity scores were not documented in our study for therapeutic plasma exchange treatment selection. 

## Figures and Tables

**Table 1:** Comparison of the characteristics, clinical and laboratory findings of the WPE group and PE.

	WPE Group *n*: 16	PE Group *n*: 22	*p*
Age months, median (range) IQR (P 25-P 75)	134 (7–214) 63–182	150 (30–210) 97–178	0.384
Sex, male, *n* (%)	10 (62.5)	13 (59)	1
Admission with hypotension, *n* (%)	2 (12.5)	9 (40.9)	0.078
Respiratory system involvement, *n* (%)	13 (81.2)	19 (86.3)	0.682
Kawasaki-like symptoms, *n* (%)	5 (31.2)	6 (27.2)	1
Laboratory parameters, median (range) IQR (P 25-P 75)			
WBC (cell/µL)	6950 (500–14,900) 4525–8300	8850 (2900–31,000) 5200–11,700	0.089
CRP (mg/L)	136 (1.5–322) 14–219	107.5 (1.7–329) 25–158	0.569
Sedimentation rate (mm/hour)	48 (6–109) 28–68	29 (4–114) 19–113	1
Procalcitonin (ng/mL)	0.6 (0,01–29) 0.38–4.2	10.3 (0,05–168) 2.59–32	**0.01**
ALT (U/L)	20 (6–107) 13–38	22 (5–381) 14–47	0.492
Ferritin (ng/mL)	236 (44–2000) 184–753	644 (27–3381) 349–1236	0.052
Fibrinogen (mg/dL)	550 (178–724) 466–590	475 (176–658) 371–568	0.200
D-dimer (µg/mL)	2.85 (0.3–19) 0.79–4.6	4 (0,36–35) 2.01–5.7	0.153
IL-6 (pg/mL)	13.3 (2,48–2000) 8–514	159 (10–1269) 53–336	0.065
Troponin I (pg/mL)	30 (0–1404) 0–98	79 (0–106,695) 40–250	0.084
NT-proBNP (pg/mL)	879 (10–1550) 529–1100	2100 (172–35,000) 750–4000	**0.0035**
Neutrophil%	84.0 (26–94) 71–88	84.5 (59–95) 80–90	0.191
Hgb (g/L)	118 (70–154) 93–131	101 (70–150) 94–118	0.308
PLT (cell/µL)	190,500.00 (700–428,000) 135,000–26,1000	163,000.00 (16,100–446,000) 80,000–281,000	0.776
Albumin (g/L)	33 (23–48) 29–40.5	30.5 (11.1–48) 25–35	0.138

Abbreviations: WBC; White blood cells, Hgb; Hemoglobin, PLT; Platelets, CRP; C-reactive protein, ALT; alanine aminotransferase, IL-6; interleukin 6, SD; standard deviation, NT-proBNP; N-terminal prohormone of brain natriuretic peptide.

**Table 2:** Comparison of the group benefitting from plasma exchange (PE group) versus the group not benefitting from it (WPE group) in terms of normalization of pathologic values, treatment modalities, hospitalization duration, and follow-up.

	WPE Group *n*: 16	PE Group *n*: 22	*p*
Time to normalization of ECG abnormalities, day, median (range) IQR (P 25–P 75)	5 (3–7) 5–5	5.5 (4–10) 4–7	0.360
Corrected QT segment values (millisecond) median IQR (25–75)	458 455–461	462 460–470	
Time to normalization of troponin I, day, median (range) IQR (P 25–P 75)	2 (1–11) 1–4	5 (1–30) 3–9	**0.044**
Mild mitral valve regurgitation (*n*) (%)	8 (50)	13 (59)	0.821
Reduced left ventricular systolic function (n) (%) Median (range) IQR (P 25–P 75)	4 (25) 24.5 (23–27) 23–26	6 (27.2) 21.5 (20–25) 20–24	1
Time to normalisation of LV systolic function median (range) IQR (P 25–P 75)	6.5 (4–45) 5–19	20 (2–50) 8–23	0.121
Intravenous immunoglobulins (*n*) (%)	13 (81.2)	19 (86.3)	0.682
Systemic steroids (*n*) (%)	10 (62.5)	19 (86.3)	0.128
Invasive mechanical ventilatory support (*n*) (%)	1 (6,2)	5 (22)	0.370
Subcutaneous enoxaparin (*n*) (%)	3 (18.7)	6 (27.2)	0.541
Length of hospital stay days, median (range) IQR (P 25–P 75)	8 ± 3.2 7 (4–15) 5.5–9.5	14.5 ± 6.8 13 (6–27) 8–20	**0.001**
Duration of follow-up, month, median (range) IQR (P 25–P 75)	6 (1–12) 3–9	6 (1–12) 3–9	0.827
Therapeutic plasma exchange sessions (*n*) Median (range) IQR (P 25–P 75)		22 5 (2–9) 5–5	

Abbreviations: ECG; electrocardiography.

## Data Availability

The data associated with the paper are not publicly available but are available from the corresponding author on reasonable request.

## Supplementary Materials

The following supporting information can be downloaded at: https://www.mdpi.com/article/10.3390/children9111640/s1, Table S1: Characteristics, symptoms, signs, comorbidities and laboratory findings of the patients; Table S2: Laboratory findings of patients at admission; Table S3: Electrocardiographic and echocardiographic findings of the patients; Table S4: Treatments given to patients other than therapeutic plasma exchange and sessions of therapeutic plasma exchange.
