# Supplementary material for: Cardiovascular Outcomes in Children with Multisystem Inflammatory Syndrome Treated with Therapeutic Plasma Exchange
Source: Children (Basel). 2022 Oct 27;9(11):1640. doi: 10.3390/children9111640 (PMC9688591; doi:10.3390/children9111640)
Supplement: Supplementary file 1 [file children-09-01640-s001.zip › Supplementary table S1.pdf]

Supplementary table S1. Characteristics, symptoms, signs, comorbidities and laboratory findings of the patients

Group A subjects	Gender	Age (months)	Symptoms and signs	Comorbidity	Laboratory	Deceased patients
1	female	60	Fever, cough		Covid PCR +	
2	male	9	Fever, hepatitis	Epilepsy	Covid PCR +	
3	male	212	Abdominal pain, vomiting, headache, tachypnea, anemia, thrombocytopenia	Chronic renal failure	Covid PCR -	
4	female	130	Fever, hypotension		Ig G +	
5	male	121	Fever, hypotension, oliguria, thrombocytopenia	Acute lymphoblastic leukemia	Covid PCR +	+
6	male	56	Fever, abdominal pain, vomiting, diarrhea		Covid PCR -	
7	male	124	Fever, abdominal pain, vomiting, diarrhea		Covid PCR +	
8	female	153	Fever, conjunctivitis, lymphadenopathy		Covid PCR +	
9	male	204	Fever, conjunctivitis, lymphadenopathy, red cracked lips		Ig G and M +	
10	male	181	Fever, abdominal pain, vomiting, diarrhea		Covid PCR +	
11	female	214	Fever, abdominal pain, vomiting, diarrhea, red cracked lips, rash, conjunctivitis		Ig G and M +	
12	male	144	Fever, thrombocytopenia		Covid PCR +	
13	male	183	Fever, abdominal pain, vomiting, diarrhea, hepatitis		Ig G and M +	
14	female	7	Fever, conjunctivitis, lymphadenopathy		Ig G and M +	
15	female	65	Fever, abdominal pain, vomiting, diarrhea, conjunctivitis, lymphadenopathy		Ig G and M +	
16	male	138	Chest pain, nausea		Ig G and M +	
Group B subjects						
1	female	119	Fever, abdominal pain, vomiting, diarrhea, hypotension, thrombocytopenia		Ig G +	
2	female	210	Fever, abdominal pain, vomiting, diarrhea, hypotension, shock, thrombocytopenia	Inflammatory bowel disease	Covid PCR -	
3	male	180	Fever, abdominal pain, vomiting, diarrhea		Covid PCR -	
4	male	142	Fever, abdominal pain, vomiting		Covid PCR -	
5	male	203	Fewer, cough, hypotension, shock		Covid PCR -	
6	male	188	Fever, abdominal pain, vomiting, diarrhea		Covid PCR -	
7	male	181	Nausea, vomiting, hypotension, shock, thrombocytopenia		Covid PCR -	+
8	female	176	Fever, abdominal pain, vomiting, diarrhea, hypotension, thrombocytopenia		Covid PCR -	
9	female	182	Fever, abdominal pain, vomiting, diarrhea		Covid PCR -	
10	male	30	Fever, cough		Covid PCR -	
11	male	175	Fever, abdominal pain, vomiting, diarrhea		Covid PCR +	
12	male	56	Fever	Hypertrophic cardiomyopathy	Ig G and M +	
13	male	97	Fever, conjunctivitis, lymphadenopathy, red cracked lips		Covid PCR +	
14	male	158	Fever, abdominal pain, vomiting, diarrhea, conjunctivitis, lymphadenopathy		Ig G and M +	
15	female	188	Fever, abdominal pain, vomiting, diarrhea, hypotension		Ig G and M +	
16	female	78	Fever, abdominal pain, vomiting, diarrhea		Ig G and M +	

17	female	85	Fever, abdominal pain, vomiting, diarrhea, conjunctivitis, lymphadenopathy		Covid PCR -	
18	male	208	Fever, chest pain		Covid PCR +	
19	female	123	Fever, abdominal pain, vomiting, diarrhea, conjunctivitis, lymphadenopathy		Ig G and M +	
20	female	123	Fever, cough		Ig G and M +	
21	male	130	Fever, abdominal pain, vomiting, diarrhea, conjunctivitis, lymphadenopathy, hypotension		Covid PCR -	
22	male	97	Fever, abdominal pain, vomiting, diarrhea, conjunctivitis, lymphadenopathy, hypotension		Ig G and M +	
