# Supplementary material for: Cardiovascular Outcomes in Children with Multisystem Inflammatory Syndrome Treated with Therapeutic Plasma Exchange
Source: Children (Basel). 2022 Oct 27;9(11):1640. doi: 10.3390/children9111640 (PMC9688591; doi:10.3390/children9111640)
Supplement: Supplementary file 1 [file children-09-01640-s001.zip › Supplementary table S2.pdf]

Supplementary table S2. Laboratory findings of patients at admission

Group A subjects	WBC	Hgb	Platelet	CRP	Sedimentation rate	Procalcitonin	Albumin	ALT	Ferritin	IL-6	Fibrinogen	D-Dimer	Pro-BNP	Troponin I
1	7600	11,9	231000	14,27		0,24	40	10	229	12	657	1,8	980	146
2	6190	15,4	249000	1,5		0,01	48	63	44	14,6	178	0,6		1404
3	4800	7	144000	5,09		2,5	30	20	1022	8,65	585	2,0		86
4	6900	10,1	428000	12,6		0,29	29	38	194	2,48	594	3,2	1100	761
5	500	7,1	700	255	14,00	8	23	107	2000	1700	408	0,3	1300	110
6	1700	8,8	152000	322	109,00	0,47	29	14	189	51	724	19,0	757	0
7	4000	10,8	263000	190	65,00	0,47	39	14	120	8	555	1,0	10	0
8	7400	11,7	129000	92	105,00	0,66	43	27	1383	7	555	3,4	1050	61
9	14900	13	200000	61	30,00	0,56	31	6	198	15	453	4,0	529	0
10	9290	12,9	313000	13,8		0,12	41	13	69		304	0,4	850	31
11	4250	13,2	141000	113	48,00	16	40	23	357	514	485	5,2	879	0
12	7000	13,4	80000	159	28,00	0,54	42	7	178		479	0,6	98	0
13	5000	15	99000	212	6,00	1,37	29	71	1283	941	491	5,3	75	22
14	11300	8,5	312000	226	48,00	29	30	20	243	2000	545	5,7	1200	0
15	7300	12	181000	159	68,00	1,59	35	38	256	3	555	2,5		30
16	9000	9,8	259000	315		5,53	25	13	483	11	625	3,5	1550	62
Group B subjects														
1	9650	10,40	80000	31		38	28,7	22	2504	356	432	5,0	2100	1218
2	31000	9,6	73000	10	29,00	168	11,1	381	721	663	177	20,0	2010	106695
3	6200	11,8	239000	25		13,5	32	14	454	222	522	2,9	1700	308
4	7700	8,8	374000	16		7,61	28	17	1143	620	568	2,7	1650	46
5	10600	11,6	405000	25		2,59	29	31	1031	10	658	2,0	1700	143
6	11700	13	281000	102	113,00	0,18	37	23	513	19	616	2,3	595	132
7	27000	7,6	61000	18		3,18	42	17	3381	280	177	21,0	2400	430
8	3500	9,4	54000	149	21,00	35	27,8	20	303	137	308	35,0	15456	200
9	2900	10	218000	156		18,8	28	28	128		176	1,1	4000	67
10	4300	13	16100	1,7	75,00	0,32	34	5	27	201	186	1,0	172	250
11	14000	7,3	446000	124	114,00	0,59	25	7	42		563	4,8	750	0
12	9600	12,5	235000	27	38,00	0,3	34	24	446		435	1,8	4628	680
13	5200	10,4	112000	158	113,00	6,9	37	10	477	92	629	4,0	536	55
14	7000	10,3	124000	190		2,68	34	22	1236	11	642	1,1	413	40
15	10350	13,9	302000	54	27,00	53	48	10	650	53	484	4,7	2400	97
16	11500	8	282000	329	23,00	31	22	268	2100	81	472	4,2	35000	91
17	2900	7	78000	268	13,00	13	24	119	1360	106	407	20,0		35
18	3700	15	146000	8	4,00	0,05	42	81	308	52	371	0,4		0
19	8100	11,8	95000	113	113,00	32	35	27	691	1269	532	12,4	3068	23
20	8000	10	180000	157	19,00	3,61	33	62	349	336	605	3,6	3800	41
21	15800	10	229000	181	114,00	14,9	20	14	639	159	478	4,0		18
22	12300	9,5	105000	199	11,00	66	24	47	1940	174	425	5,7	18000	58
