# Supplementary material for: Cardiovascular Outcomes in Children with Multisystem Inflammatory Syndrome Treated with Therapeutic Plasma Exchange
Source: Children (Basel). 2022 Oct 27;9(11):1640. doi: 10.3390/children9111640 (PMC9688591; doi:10.3390/children9111640)
Supplement: Supplementary file 1 [file children-09-01640-s001.zip › Supplementary table S3.pdf]

Supplementary table S3. Electrocardiographic and echocardiographic findings of the patients

Group A subjects	ECG abnormalities and QTc values (millisecond)	Echocardiographic findings	Mitral valve regurgitation	Reduced left ventricle systolic function defined as fractional shortening %	Aortic valve regurgitation
1			Mild		
2					
3	Low QRS voltage	Pericardial effusion and left ventricular hypertrophy			
4			Mild	27	
5	QT segment prolongation QTc: 461	Pericardial effusion and dilatation of left main coronary artery		24	
6			Mild		
7			Mild		Mild
8	QTc prolongation, negative T waves in V_5 and V_6 leads		Mild	23	
9			Mild		
10	Negative T waves in V_5 and V_6 leads		Mild		
11	Low QRS voltage	Dilatation of left main coronary and left anterior descending artery	moderate		
12	Low QRS voltage				
13					
14	ST segment depression/elevation		Mild		Mild
15					
16	QT segment prolongation QTc: 455	Pericardial effusion	moderate	25	
Group B subjects					
1	Low QRS voltage		Mild	20	
2	Low QRS voltage, QT segment prolongation QTc: 458		Mild	22	Mild
3			Mild	24	
4	First degree block	Increased perivascular echogenicity of the left main coronary artery	Mild		
5	ST segment depression/elevation		Mild		
6	QT segment prolongation QTc: 462		Mild		
7		Left ventricular hypertrophy	Mild	20	
8					
9					
10		Increased perivascular echogenicity of the left main coronary artery			
11			Mild		
12	Left ventricular hypertrophy		Moderate		Mild
13					
14					

15	Low QRS voltage, negative T waves in V_5 and V_6 leads		Mild	21	
16			Mild		
17			Mild		
18		Asymmetrical septum hypertrophy			
19					
20	Low QRS voltage, QT segment prolongation QTc: 470	Dilatation of right main coronary artery	Moderate		
21	QT segment prolongation QTc: 460	Left ventricular hypertrophy	Mild		
22	QT segment prolongation QTc: 475		Mild	25	Mild
