# Supplementary material for: Cardiovascular Outcomes in Children with Multisystem Inflammatory Syndrome Treated with Therapeutic Plasma Exchange
Source: Children (Basel). 2022 Oct 27;9(11):1640. doi: 10.3390/children9111640 (PMC9688591; doi:10.3390/children9111640)
Supplement: Supplementary file 1 [file children-09-01640-s001.zip › Supplementary table S4.pdf]

Supplementary table S4. Treatments given to patients other than therapeutic plasma exchange and sessions of therapeutic plasma exchange

Group A Subjects	Intravenous immunoglobulin	Systemic steroids	Subcutaneous enoxaparin treatments	Invasive mechanical ventilation support	Tocilizumab	Anakinra	Extracorporeal membrane oxygenation	Number of sessions of therapeutic plasma exchange
1								
2	+							
3		+						
4	+	+	+					
5	+	+		+				
6	+	+						
7	+							
8		+	+					
9	+	+						
10	+							
11	+	+						
12	+	+						
13	+	+						
14	+							
15	+							
16	+	+	+					
Group B subjects								
1	+	+			+		+	4
2	+	+			+	+	+	8
3	+	+						5
4	+	+						5
5	+							2
6	+	+						5
7	+	+			+	+	+	5
8	+	+		+				6
9								5
10								6
11	+	+						5
12	+	+	+					9
13	+	+	+					5
14	+	+						5
15	+	+	+					5
16	+	+		+				5
17	+	+		+				9
18	+	+						3
19	+	+						5
20		+	+	+				5
21	+	+	+					5
22	+	+	+	+				5
